# First-Principles Study on Possible Half-Metallic Ferrimagnetism in Double Perovskites Pb_2_XX′O_6_ (X = Ti, Zr, Hf, V, Nb and Ta, X′ = Tc, Ru, Os and Rh)

**DOI:** 10.3390/ma15093311

**Published:** 2022-05-05

**Authors:** Bo-Yu Chen, Po-Han Lee, Yin-Kuo Wang

**Affiliations:** 1 Affiliated Senior High School of National Taiwan Normal University, Taipei 10658, Taiwan; matt930929@gmail.com; 2 Department of Electro-Optical Engineering, National Taipei University of Technology, Taipei 10608, Taiwan; 3 Center for General Education and Department of Physics, National Taiwan Normal University, Taipei 10610, Taiwan

**Keywords:** double perovskites, first-principle calculation, half metal, ferrimagnetic state

## Abstract

Pb-based double perovskite compounds with chemical formula Phey have abundant physical properties in the spintronic field. Among all the features, the spin interaction of half-metallic (HM) is regarded as an important performance measure because of its high potential in spintronic devices. In this research study, we calculate density of state (DOS) to investigate possible half-metal candidates by executing structural optimization based on the method of generalized gradient approximation (GGA) and strong correlation effect (GGA + U). Furthermore, following the earlier methods by calculating and comparing energy difference of various compounds with the four initial magnetic states: ferromagnetic, ferrimagnetic, antiferromagnetic and nonmagnetic, we can determine which magnetic state is more stable. Results indicate that there are 13 possible ferrimagnetic HM candidates in these combinations, including Pb_2_NbTcO_6_, Pb_2_TaTcO_6_, Pb_2_TiRuO_6_, Pb_2_ZrRuO_6_, Pb_2_HfRuO_6_, Pb_2_VRuO_6_, Pb_2_NbRuO_6_, Pb_2_TadRuO_6_, Pb_2_ZrOsO_6_, Pb_2_HfOsO_6_, Pb_2_VOsO_6_, Pb_2_ZrRhO_6_ and Pb_2_HfRhO_6_ under GGA and GGA + U schemes. The stability of analysis by analyzing the energy gap illustrates that all 13 possible candidates are half metals and ferrimagnetic states, so our studies could provide guidelines for scientists to fabricate new double perovskites in future.

## 1. Introduction

Half metals (HMs) are potential and popular materials in the field of spintronics device research [1,2,3,4,5,6,7,8] owing to their function of inducing 100% spin polarization. With an aim to discover more HMs, the group of double perovskites is an ideal selection because they account for an enormous majority of known HMs, including Sr_2_FeMoO_6_ [5,9,10,11], Sr_2_FeReO_6_ [9,12], La_2_VTcO_6_ [12], La_2_VCuO_6_ [12], La_2_MoTcO_6_ [13], La_2_WReO_6_ [14], BiPbVRuO_6_ [15], Bi_2_CrCoO_6_ [16] and Bi_2_FeNiO_6_ [16], mixed valance perovskite structures manganese oxide Ln_0.5_Ca_0.5_MnO_3_ [17] and Ln_0.7_Sr_0.3_MnO_3_ [18,19], spinel FeCr_2_S_4_ [20,21] and Mn-doping GaAs [22,23]. On the other hand, the topic of magnetism has also received significant attention in the discussion of double perovskites family, the magnetic state ranging from antiferromagnetic [24,25,26,27,28,29] to ferrimagnetic [30,31,32] and ferromagnetic [33,34,35]. Among them, ferrimagnetic materials are widely used in non-volatile memory devices such as hard drives, which utilize their ability to easily switch the spins of electrons and be magnetized.

Due to the structural and compositional flexibility of the double perovskites structure, many researchers are continually disclosing new HM materials from the group of double perovskites A_2_XX′O_6_, where A is a relatively large cation [36,37,38], and X and X′ are metal ions. It is anticipated that study of the replacement of the large lead(II) cation in A site element [39] could provide opportunities to find stable HM candidates in related research, because Pb^2+^ has a suitable size to be combined with smaller X and *X*′ site cations to satisfy the tolerance criterion (*t*) noted by Goldschmidt [40], with *t* having a value close to unity for stable perovskite structures. In this regard, some compounds of Pb_2_XX′O_6_ in previous studies were experimentally synthesized [41,42,43], in which there are indeed some HM materials, i.e., Pb_2_TcReO_6_ [44], Pb_2_MoOsO_6_ [44], Pb_2_FeRuO_6_ [45], Pb_2_FeMoO_6_ [45], Pb_2_CrRuO_6_ [46] and Pb_2_CrOsO_6_ [46]. It is evident that the choice to select X and X′ allows one to decide the physical properties of double perovskites because of their cation size and valance distribution of *d* (or *f*) orbitals [47,48,49].

However, in the strictest sense, perfect half-metallicity is limited to ideal crystals at zero Kelvin temperature; real HMs mainly exhibit dramatic decreases in the spin polarization due to thermal effects and intrinsic crystal and surface imperfection [50], which are ignored by the calculation of density functional theory (DFT) [51]. Nevertheless, for conquering the implicated synthesis processes of double perovskite compounds, some researchers indeed found good agreement of experimental synthesized results about HMs with the theory predictions such as Sr_2_FeReO_6_ [52,53]. For the magnetic property of Pb_2_MnWO_6_ [54], Ivanov et al. used DFT-based calculations to predict the presence of a low-temperature magnetic ordering, which matches their experimental results. Consequently, the DFT calculation also makes it possible to predict the properties of some compounds in the condition of zero Kelvin temperature, and provides limited but useful information at finite temperature.

Following the solid work that shows that some cyclical behaviors could be determined by the XX′ pairs [55,56], we will focus here on the transition metal combination of IVB/VB group and VIIB/VIIIB group and attempt to thoroughly investigate potential HM candidates in the group of Pb_2_XX′O_6_. The method with which we calculate these double perovskites is based on DFT and the procedure is shown as follows: First, we optimize our structure by the method of generalized gradient approximation (GGA) [57]. After that, through the process of optimization, we determine whether or not the material is an HM candidate by two rules: one is the integer spin magnetic moment from compounds, and the other one is the energy gap existing in the single-side channel provided by density of states (DOS). In other words, the band gap of either spin-up or spin-down is indeed observed and exists in the single channel. Next, the consideration of strong correlation effect (GGA + U) [58,59,60] is also checked to make sure that there is stability of the energy gap, which is then computationally convenient for accurate calculations of electronic structures. Last, the same method is executed repeatedly with four initial states, i.e., ferromagnetic (FM), ferrimagnetic (FiM), antiferromagnetic (AF) and nonmagnetic (NM). Finally, magnetic states of these double perovskites are then verified by the energetic comparison of these results.

## 2. Materials and Methods

**Figure 1:**
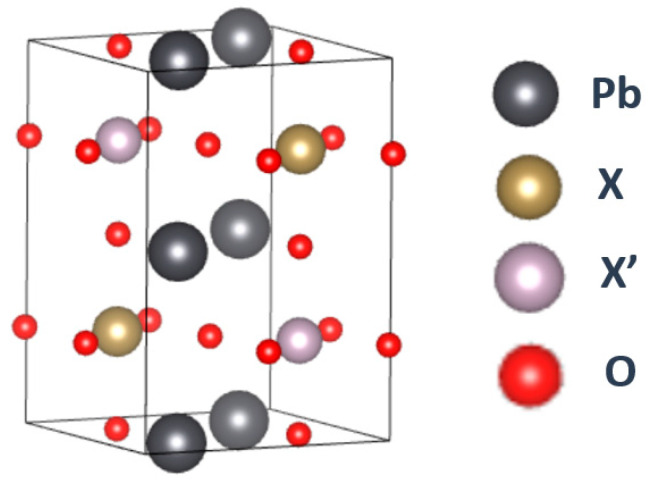
An ideal ordered double perovskite structure of Pb_2_XX′O_6_.

**Figure 2:**
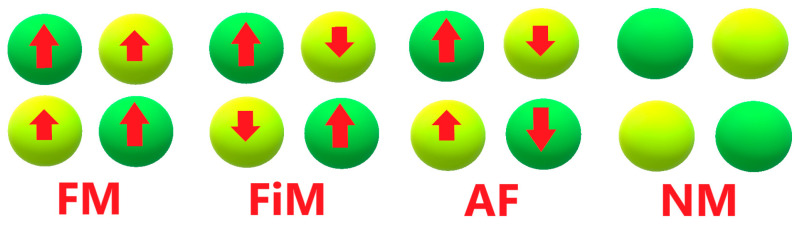
The schematic diagram of four magnetic states: FM, FiM, AF and NM.

The Pb-based double perovskites consist of Pb, IVB/VB transition metals (Ti, Zr, Hf, V, Nb and Ta) paired with VIIB/VIIIB transition metals (Tc, Ru, Os and Rh) and oxygen, as shown in Figure 1. In total, there are 24 kinds of compounds counted upon which structural optimization is executed in order to check which one is the more stable HM material candidate. Furthermore, this study begins with the four types of initial magnetic states, i.e., ferromagnetic (FM), ferrimagnetic (FiM), antiferromagnetic (AF) and nonmagnetic (NM) for each compound, as shown in Figure 2. Accordingly, based on results, comparison between each state can provide information about the stable situation for all compounds. Lastly, density of states (DOS) is analyzed for d-orbital electrons to confirm not only magnetic but also half-metallic properties. By doing so, HM candidates could be picked up through protracted and complex processes. Last, all candidates are examined in consideration of the strong correlation effect (GGA + U).

Next, we determine whether the structures are stable or not after full structural optimization, implying two structures being discussed. One is the tetragonal structure (space group of I4/mmm, No. 139), and it is made of two non-equivalent types of oxygen atoms, in which the locations of O_1_ atoms are on the z-axis and there are four O_2_ atoms existing on the xy-plane, as shown in Figure 1. Accordingly, they are the cases of F(i)M state. The other one is the tetragonal structure (space group of P4/mmm, No. 123) with non-equivalent types of oxygen atoms, in which the angle of X-O_1_-X′ is maintained at 180° and the angle of X-O_2_-X′ has been changed a little but is still near 180° during the process of structural optimization. This means that the symmetry reduction is deemed rather minor and the c/a ratio is very close to the value of $\sqrt{2}$. These features could be found in the AF state.

In the FM and FiM states, each X and X′ ion has similar spin states (that is, (X, X, X′, X′) = (m, m, m′, m′) = FM or (m, m, −m′, −m′) = FiM), which can cause the assumption of the half-metallicity of the double perovskite. By the self-consistent process, most of the initial FM and FiM states all converge into one of the states. In the AF states, the spin state of (X, X, X′, X′) can be noted as (m, −m, m′, −m′). The induced equivalence in the charge is Q↑[X(X′)] = Q↓[X(X′)], which can be observed from the symmetry of the spin-up and spin-down in the total figure of density of state (DOS). No spin polarization is observed in the NM state. Calculation results for all four magnetic phases are performed to find the most stable magnetic phase. (However, when we put spin polarization into consideration, the calculation results show that the compounds become more stable.) The self-consistent process with high convergence requirement is also performed to guarantee the accuracy of the result.

In this research, we present electronic structure calculations with generalized gradient approximation (GGA) plus on-site coulomb interaction (GGA + U). Structural optimization calculations are carried out through the full-potential projector-augmented wave [61] (PAW) method by using the code of the Vienna Ab Initio Simulation Package (VASP) [62,63,64] to determine the theoretical lattice constraints and atomic positions. The calculation for the Brillouin zone is conducted using 8×8×6 Monkhorst–Pack k-grid sampling. The cut-off energy of the plane wave basis is set to 450 eV. The energy convergence criteria for the full structure optimization and self-consistent calculations are set to $1\times 10^{-5}$ and $1\times 10^{-7}$ eV, respectively. The Wigner–Seitz radius of the Pb atom is set as 3.3 atomic units (a.u.), 1.6 a.u. for O atom and 2.7 a.u. for X(X′) ion. For the final and equilibrium structures, the forces and stresses acting on all the atoms are less than 0.3 eV/Å and 0.9 kBar, respectively.

## 3. Results and Discussion

**Figure 3:**
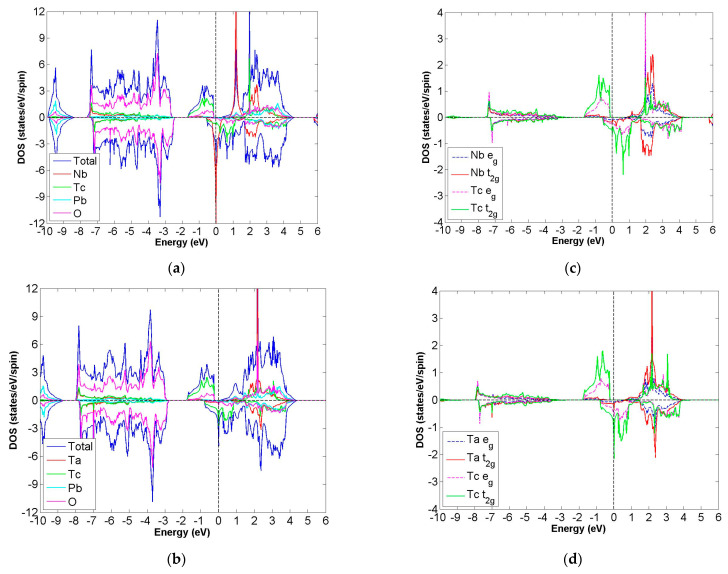
Based on the GGA calculation, the calculated total and partial DOS values of (**a**) Pb_2_NbTcO_6_ and (**b**) Pb_2_TaTcO_6_ and the partial DOS of e_g_ and t_2g_ spin orbitals for (**c**) Nb and Tc and (**d**) Ta and Tc.

**Figure 4:**
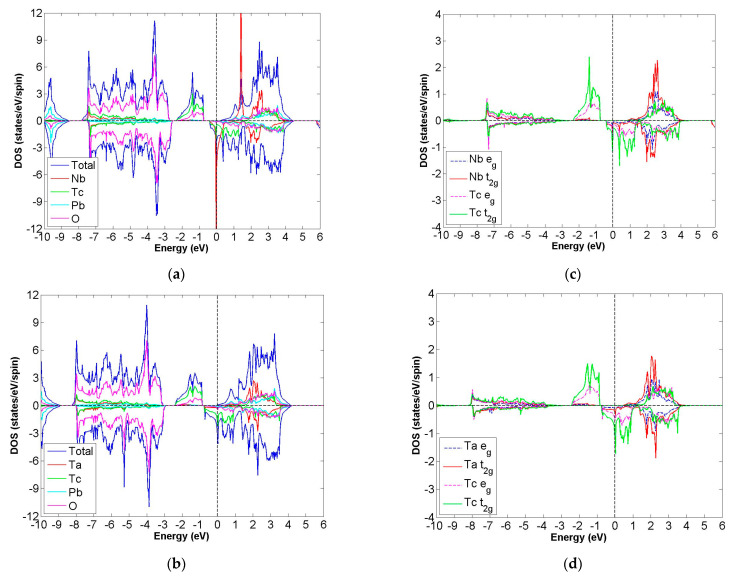
Under GGA + U (U of Nb, Ta and Tc set up as 2) schemes, the calculated total and partial DOS values of (**a**) Pb_2_NbTcO_6_, (**b**) Pb_2_TaTcO_6_ and the partial DOS of e_g_ and t_2g_ spin orbitals for (**c**) Nb and Tc and (**d**) Ta and Tc.

**Figure 5:**
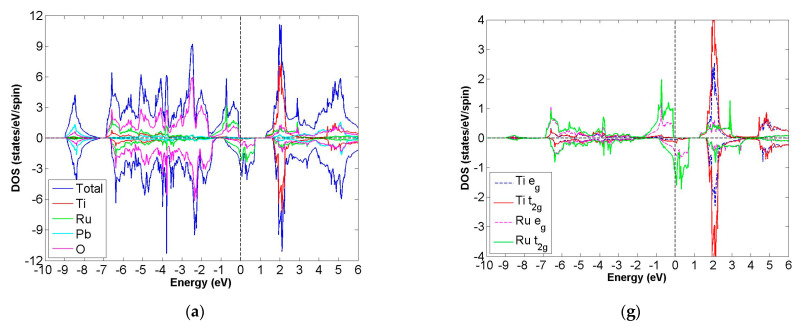

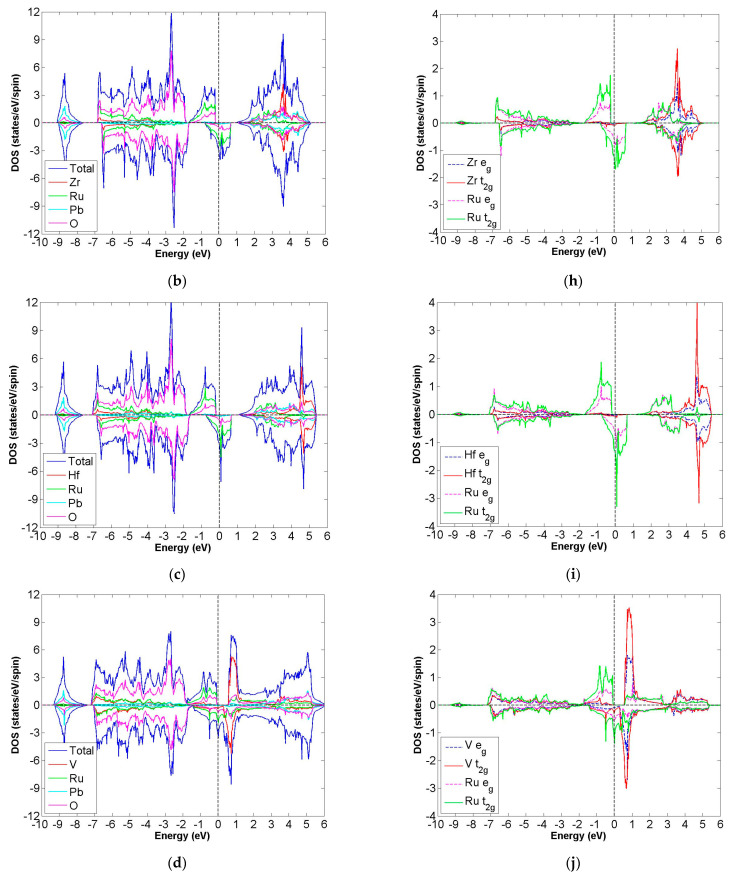

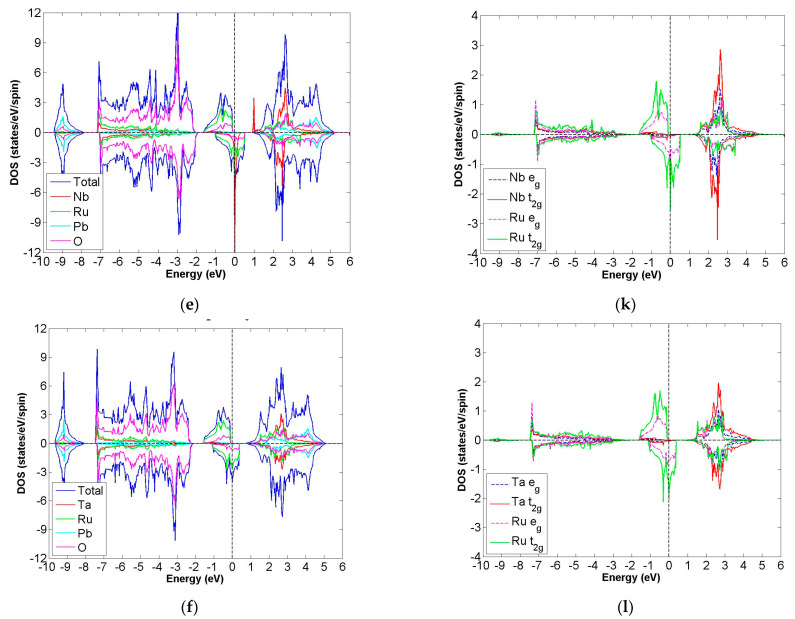
Based on the GGA calculation, the calculated total and partial DOS values of (**a**) Pb_2_TiRuO_6_, (**b**) Pb_2_ZrRuO_6_, (**c**) Pb_2_HfRuO_6_, (**d**) Pb_2_TiRuO_6_, (**e**) Pb_2_ZrRuO_6_ and (**f**) Pb_2_HfRuO_6_ and partial DOS of e_g_ and t_2g_ spin orbitals for (**g**) Ti and Ru, (**h**) Zr and Ru, (**i**) Hf and Ru, (**j**) V and Ru, (**k**) Nb and Ru, (**l**) Ta and Ru.

**Figure 6:**
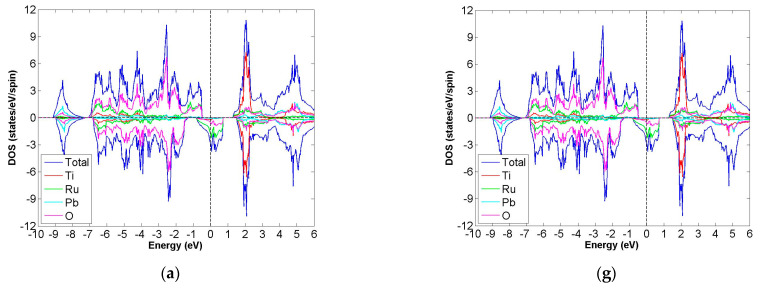

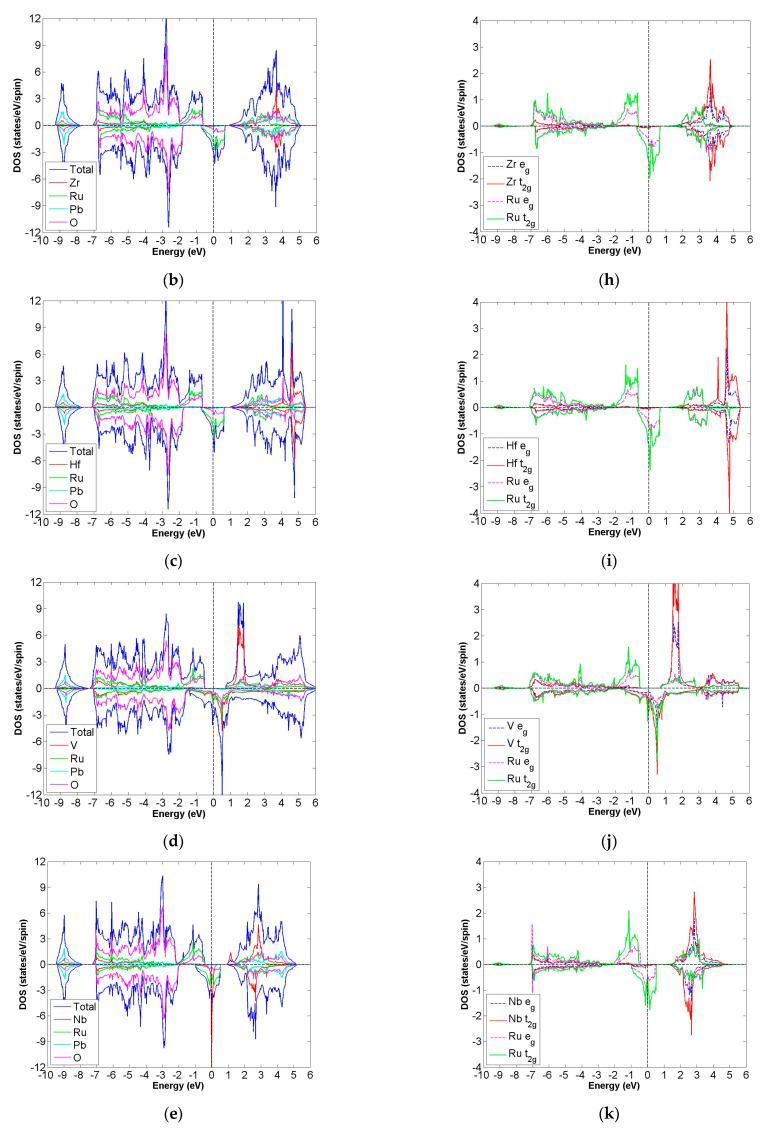

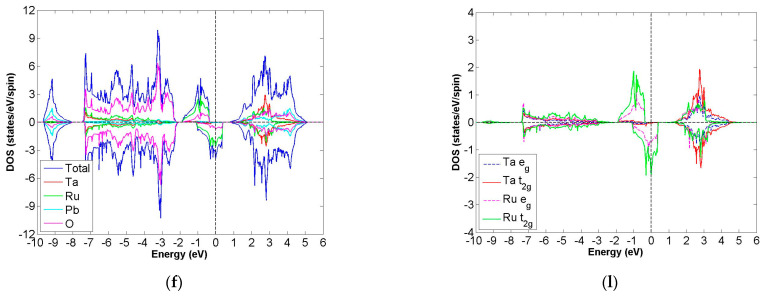
Under GGA + U (U of Ti, Zr, Hf, V, Nb, Ta and Ru set up as 2) schemes, the calculated total and partial DOS values of (**a**) Pb_2_TiRuO_6_, (**b**) Pb_2_ZrRuO_6_, (**c**) Pb_2_HfRuO_6_, (**d**) Pb_2_VRuO_6_, (**e**) Pb_2_NbRuO_6_ and (**f**) Pb_2_TaRuO_6_ and the partial DOS of e_g_ and t_2g_ spin orbitals for (**g**) Ti and Ru, (**h**) Zr and Ru, (**i**) Hf and Ru, (**j**) V and Ru, (**k**) Nb and Ru, (**l**) Ta and Ru.

**Figure 7:**
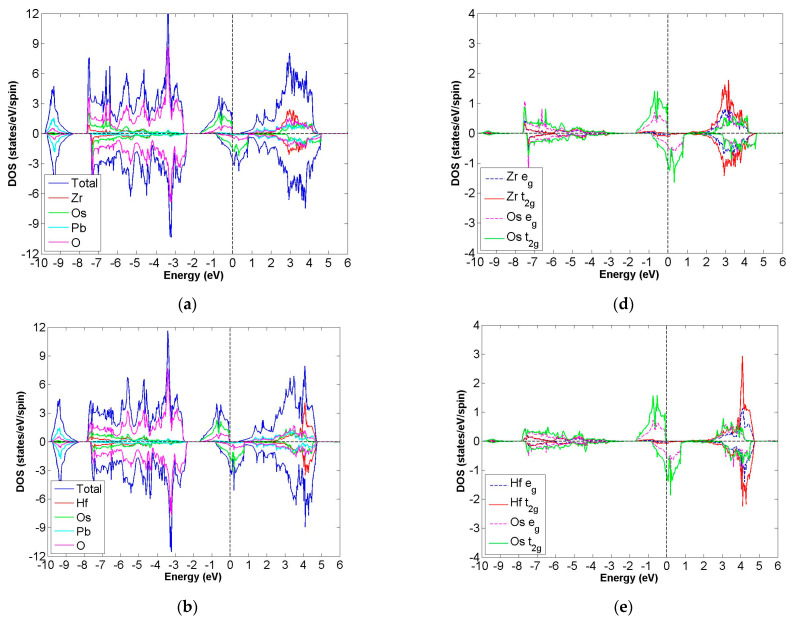

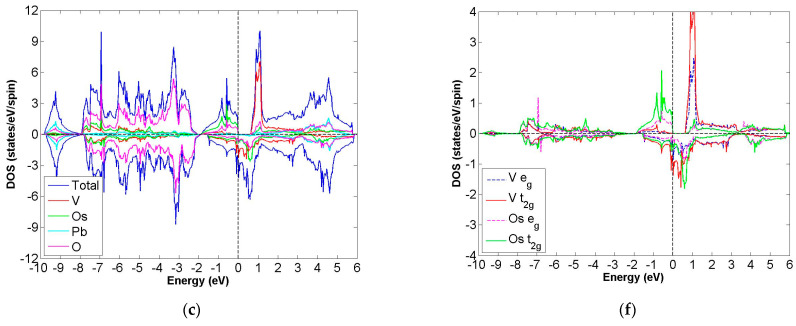
Based on the GGA calculation, the calculated total and partial DOS values of (**a**) Pb_2_ZrOsO_6_, (**b**) Pb_2_HfOsO_6_ and (**c**) Pb_2_VOsO_6_ and the partial DOS of e_g_ and t_2g_ spin orbitals for (**d**) Zr and Os, (**e**) Hf and Os and (**f**) V and Os.

**Figure 8:**
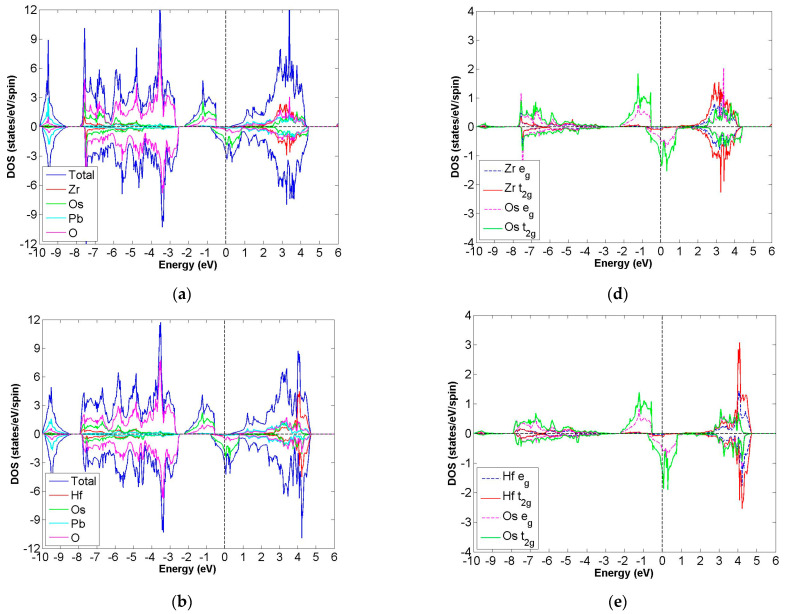

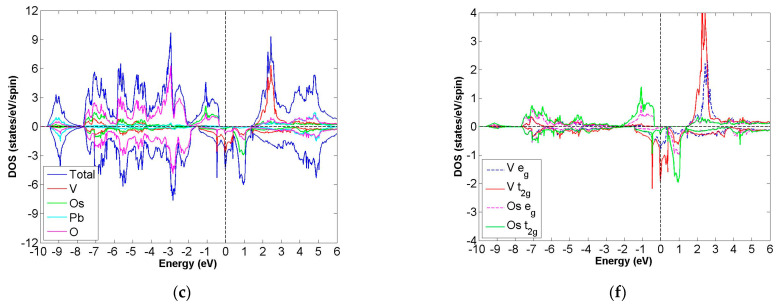
Under GGA + U (U of Zr, Hf, V and Os set up as 2) schemes, the calculated total and partial DOS values of (**a**) Pb_2_ZrOsO_6_, (**b**) Pb_2_HfOsO_6_ and (**c**) Pb_2_VOsO_6_ and the partial DOS of e_g_ and t_2g_ spin orbitals for (**d**) Zr and Os and (**e**) Hf and Os and (**f**) V and Os.

**Figure 9:**
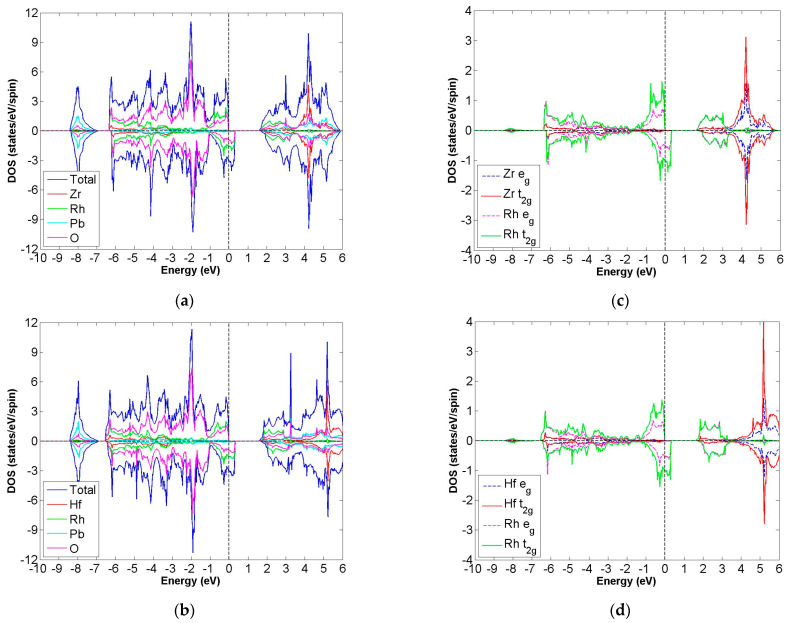
Based on the GGA calculation, the calculated total and partial DOS values of (**a**) Pb_2_ZrRhO_6_ and (**b**) Pb_2_HfRhO_6_ and partial DOS of e_g_ and t_2g_ spin orbitals for (**c**) Zr and Rh and (**d**) Hf and Rh.

**Figure 10:**
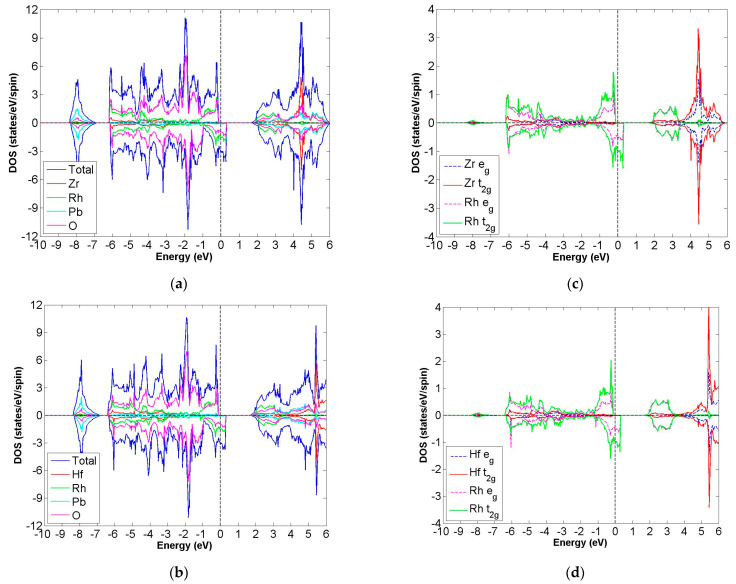
Under GGA + U (U of Zr, Hf and Rh set up as 2) schemes, the calculated total and partial DOS values of (**a**) Pb_2_ZrRhO_6_, (**b**) Pb_2_HfRhO_6_ and the partial DOS of e_g_ and t_2g_ spin orbitals for (**c**) Zr and Rh and (**d**) Hf and Rh.

After calculation under the GGA scheme, we find that 13 out of the 24 compounds in the Pb_2_XX′O_6_ are categorized as HMs, including Pb_2_NbTcO_6_, Pb_2_TaTcO_6_, Pb_2_TiRuO_6_, Pb_2_ZrRuO_6_, Pb_2_HfRuO_6_, Pb_2_VRuO_6_, Pb_2_NbRuO_6_, Pb_2_TaRuO_6_, Pb_2_ZrOsO_6_, Pb_2_HfOsO_6_, Pb_2_VOsO_6_, Pb_2_ZrRhO_6_ and Pb_2_HfRhO_6_. Based on these results, Tc, Ru, Os and Rh are suitable for substitute X′ site element, and we follow this order to systemically discuss all possible HM candidates. For clearer description with the GGA scheme, Figure 3a–d describe the DOS of Pb_2_NbTcO_6_, Pb_2_TaTcO_6_ and PDOS of d-orbital in Pb_2_NbTcO_6_ and Pb_2_TaTcO_6_, respectively. In comparison with these figures, Figure 4a–d illustrate the same compounds just under the GGA + U scheme. Following the figure arrangement of GGA and GGA + U, Figure 5a–l and Figure 6a–l, Figure 7a–f and Figure 8a–f and Figure 9a–d and Figure 10a–d illustrate the DOS and PDOS of Pb_2_XRuO_6_ (X = Ti, Zr, Hf, V, Nb and Ta), Pb_2_XOsO_6_ (X = Zr, Hf and V) and Pb_2_XRhO_6_ (X = Zr and Hf), respectively.

### 3.1. FiM-HM Compounds: Pb_2_XTcO_6_ (X = Nb and Ta)

In Table 1, it shows the energy difference between AF and FiM states; note that ΔE = FiM—AF and their values are −21 and −96 meV/f.u. for Pb_2_NbTcO_6_ and Pb_2_TaTcO_6_, respectively. Furthermore, with the GGA + U scheme, the values of ΔE decrease to −26 and −178 meV/f.u., indicating a more stable state of FiM for these compounds, thus illustrating direct evidence that these materials are ferrimagnetic. Table 2 lists all the energy of AF and FiM states in detail. 

As shown in Figure 3a,b, the band gaps of Pb_2_NbTcO_6_ and Pb_2_TaTcO_6_ occur in the spin-up channel, while some electrons occupy the Fermi level of the spin-down channel, which provides obvious evidence for HM materials. As to the second indicator for half-metal compound, it is the integer value of m_tot_. Here, the values of m_tot_ are 2.000 μ_B_/f.u. in the case of Pb_2_NbTcO_6_ and Pb_2_TaTcO_6_, which illustrates HM property for these compounds. In addition, Pb_2_TaTcO_6_ still maintains the possibility of being HM material under the GGA + U scheme with a little fluctuation of magnetism because the m_tot_ value of Pb_2_TaTcO_6_ changes from 2.000 to 2.011 μ_B_/f.u.

However, these variations (+U, the coulomb interaction potential) do not change the magnetic states of compounds; instead, the tendency of being FiM material under the GGA + U scheme is more stable than that under the GGA scheme. In addition, under these two schemes, the distributions of electrons whose energy is higher than Fermi energy are almost the same.

Next, we discuss the electronic configuration of the two compounds. For Pb_2_NbTcO_6_, the ideal electronic distributions of Nb and Tc we expected are Nb^5+^ (3d^10^4s^2^4p^6^: t^0^_2g_e^0^_g_) at S = 0 and Tc^3+^(4d^4^5s^0^:t^4^_2g_e^0^_g_) at S = 1. From the result of calculations with the GGA method, the electron distributions are Nb^2.9+^(4d^2.1^) and Tc^2.4+^(4d^4.6^), and those with the GGA + U scheme are Nb^3.1+^(4d^1.9^) and Tc^2.5+^(4d^4.5^).

In the case of Pb_2_TaTcO_6_, the valance states are Ta^5+^ (4f^14^5d^0^6s^0^:t^0^_2g_e^0^_g_) at S = 0 and Tc^3+^ (4d^4^5s^0^:t^4^_2g_e^0^_g_) at S = 1. After the GGA calculation, we find that the actual valance states of Ta and Tc are 2.1 and 4.6, as shown in Table 1. We notice Ta^2.8+^ (5d^2.2^) and Tc^2.4+^ (4d^4.6^). With the GGA + U scheme, the *d* orbital electrons of Ta and Tc are 2.2 and 4.5, which imply the electronic configurations are Ta^2.9+^(5d^2.1^) and Tc^2.4+^(4d^4.6^).

The significant feature of this group is Pb_2_TaTcO_6_, also illustrating half-metallic property under the GGA scheme and GGA + U scheme, while it is considered to be a little fluctuation of magnetism under the GGA + U scheme. This phenomenon may be confirmed through experiments in the future; our calculations just provide an accurate answer about whether this compound is a possible HM candidate.

### 3.2. FiM-HM Compounds: Pb_2_XRuO_6_ (X = Ti, Zr, Hf, V, Nb and Ta)

In the case of Pb_2_XRuO_6_, all combinations can be categorized into the half-metal family, namely Pb_2_TiRuO_6_, Pb_2_ZrRuO_6_, Pb_2_HfRuO_6_, Pb_2_VRuO_6_, Pb_2_NbRuO_6_ and Pb_2_TaRuO_6_. All of them are FiM materials, indicated by ΔE values, which are −43, −55, −50, −18, −49 and −22 meV/f.u. for these compounds, respectively. With examination of the cases under the GGA + U scheme, they also remain in the same magnetic state; ΔE values of them are −24, −106, −92, −139, −115 and −60 meV/f.u., respectively. As a result, all compounds in this group are FiM materials under these schemes.

As seen in Table 1, m_tot_ for Pb_2_TiRuO_6_, Pb_2_ZrRuO_6_ and Pb_2_HfRuO_6_ are 2.000 μ_B_/f.u., but those of the others are maintained at 1.000 μ_B_/f.u. Even though their values are not the same, all of them are FiM-HM materials because their m_tot_ are integers except zero. The other evidence for half-metal property is provided by Figure 5a–l. The band gaps of these compounds only occur in the spin-up channel, while the spin-down channel is conductive.

When we monitor electrons near Fermi energy, the distribution of Pb_2_TiRuO_6_ is like the cases of Pb_2_ZrRuO_6_ and Pb_2_HfRuO_6_, and DOS of Pb_2_VRuO_6_ is analogous to those of Pb_2_NbRuO_6_ and Pb_2_TaRuO_6_. The difference between them is the DOS of X site element in the spin-up channel. PDOS of Ti, Ru and Hf are almost concentrated, ranging from about 2 to 5 eV, and in the case of V, Nb and Ta, PDOS of them ranges from about 1 to 5 eV. When compared with Table 1, this phenomenon leads to the band gap values of Pb_2_VRuO_6_, Pb_2_NbRuO_6_ and Pb_2_TaRuO_6_ being 1.35, 1.18 and 1.25 eV, larger than those of Pb_2_VRuO_6_, Pb_2_NbRuO_6_ and Pb_2_TaRuO_6_, which are 0.62, 1.00 and 0.85 eV, respectively.

When using the nominal valance states, the ordered double perovskites point to the state of Pb_2_^2+^(XX′)^8+^O_6_. In this paragraph, we will discuss the ideal valance configurations of Pb_2_TiRuO_6_, Pb_2_ZrRuO_6_ and Pb_2_HfRuO_6_, and then compare them with the calculation results of both GGA and GGA + U schemes. First, the transition metal elements (X, X′) of Pb_2_TiRuO_6_ are Ti and Ru, in which Ti and Ru have valance configurations of Ti^4+^(3d^0^) and Ru^4+^(3d^4^), so the valance states are Ti^4+^(3d^0^:t^0^_2g_e^0^_g_) at S = 0 and Ru^4+^(4d^4^4s^0^: t^4^_2g_e^0^_g_) at S = 1. Nevertheless, the valance configurations were simplified based on the ideal ionic model. Therefore, the electronic number of elements that would be redistributed in consideration of the process of hybridization among Ti 3d, Ru 4d and oxygen 2p orbitals is ignored in this model. The redistribution results of the electronic number indicate Ti and Ru having total electron numbers of 2.0 and 6.0 for d orbitals, showing the valance states of Ti^2+^(3d^2^) and Ru^2+^(4d^6^). In the consideration of GGA + U schemes, Ti and Ru have 1.9 and 6 electrons for d orbitals, so the valance states are Ti^2.1+^(3d^1.9^) and Ru^2+^(4d^6^).

In the ionic picture of Pb_2_ZrRuO_6_, the formal valance of ZrRu is +8, and the electronic configurations are Zr^4+^ (4d^0^:t^6^_2g_e^4^_g_), S = 0 and Ru^4+^(4d^4^4s^0^: t^4^_2g_e^0^_g_) at S = 1. According to the calculation results, we found Zr and Ru total electron numbers of 1.5 and 6.0 for d orbitals, showing the valance states of Zr^2.5+^(4d^1.5^) and Ru^2+^(4d^6^); in the case of Pb_2_HfRuO_6_, the electronic configurations are Hf^4+^(5d^0^:t^0^_2g_e^0^_g_) at S = 0 and Ru^4+^(4d^4^4s^0^: t^4^_2g_e^0^_g_) at S = 1, and the actual situations are Hf^2.4+^(4d^1.6^) and Ru^2+^(4d^6^). With the GGA+U scheme, as in the case of GGA, the valance states are Zr^2.5+^(4d^1.5^) and Ru^2+^(4d^6^), just differences in Hf^2.5+^(4d^1.5^).

In the ionic picture of Pb_2_VRuO_6_, the formal valance of VRu is +8, and the electronic configurations are V^5+^ (3d^0^:t^0^_2g_e^0^_g_), S = 0 and Ru^3+^(4d^5^4s^0^: t^5^_2g_e^0^_g_) at S = 1/2. According to the calculation result, we found V and Ru total electron numbers of 3.6 and 6.1 for d orbitals, showing the valance states of V^1.4+^(3d^3.6^) and Ru^1.9+^(4d^6.1^). With GGA + U scheme, there are 3.6 and 6.1 electrons for d orbitals of V and Ru, which implies the valance states are V^1.5+^(3d^3.5^) and Ru^1.9+^(4d^6.1^).

In the case of Pb_2_NbRuO_6_, the electron configurations are Nb^5+^(3d^10^4s^2^4p^6^: t^0^_2g_e^0^_g_) at S = 0 and Ru^3+^(4d^5^4s^0^: t^5^_2g_e^0^_g_) at S = 1/2. The result provides that the valance states of double perovskites are Nb^2.8+^(4d^2.2^) and Ru^1.9+^(4d^6.1^) with the GGA scheme. On the other hand, the distribution with the GGA + U scheme of electron number is Nb^3.0+^(4d^2^) and Rh^1.9+^(4d^6.1^).

Last, Pb_2_TaRuO_6_ consists of Ti and Ru, which have valance configurations Ta^5+^(4f^14^5d^0^6s^0^:t^0^_2g_e^0^_g_) at S = 0 and Ru^4+^(4d^4^4s^0^: t^4^_2g_e^0^_g_) at S = 1. The redistribution of electronic number provided by calculation with the GGA method indicates Ta and Ru having total electron numbers of 2.2 and 6.2 for d orbitals, showing the valance states of Ta^2.8+^(5d^2.2^) and Ru^1.8+^(4d^6.2^). In the consideration of GGA + U schemes, Ta and Ru have 2.1 and 6.2 electrons for d orbitals, so electron distributions are Ta^2.9+^(5d^2.1^) and Ru^2+^(4d^6.2^).

In this group, here, the most important result is that these combinations provide an assortment of HM candidates, with all double perovskites including the Ru element presenting half-metallic property. In the aspect of magnetic state, these six compounds belong to the FiM phase category, and ΔE of them are significant enough to determine it.

### 3.3. FiM-HM Compounds: Pb_2_XOsO_6_ (X = Zr, Hf and V)

After full structural optimization, the magnetic states for Pb_2_ZrOsO_6_, Pb_2_HfOsO_6_ and Pb_2_VOsO_6_ converge to FiM state. Under the GGA scheme, ΔE for these compounds are −27, −28 and −30 meV/f.u., and with the GGA + U scheme, they decrease to −112, −97 and −309 meV/f.u., respectively. All the evidence illustrates a greater tendency for the FiM state.

As seen in Table 1, m_tot_ for Pb_2_ZrOsO_6_ and Pb_2_HfOsO_6_ is 2.000 μ_B_/f.u., and m_tot_ for Pb_2_VOsO_6_ is 1.000 μ_B_/f.u. Table 1 also shows the energy gaps for these compounds, which are 0.38, 0.47 and 0.65 eV. When we refer to Figure 7a,b, a few electrons occupy the energy range of 0.5 to 1 eV, so this phenomenon makes the band gaps of the group compounds narrower than others. These results also provide direct evidence indicating that these compounds belong to the FiM-HM family.

Next, we investigate ideal electron configuration with covalent electron theory. For Pb_2_ZrOsO_6_, it has electron configuration Zr^4+^(4d^0^:t^6^_2g_e^4^_g_) at S = 0 and Os^4+^(4f^14^5d^4^5s^0^:t^4^_2g_e^0^_g_) at S = 1. Table 1 shows the electron configurations of Pb_2_ZrOsO_6_, describing Zr^2.5+^(4d^1.5^) and Os^2.6+^(5d^5.4^) under the GGA scheme, which indicates that Zr and Os contribute almost the same electron numbers for the bond. With the GGA + U scheme, the results are similar to previous results; with the GGA scheme, the electron configurations are also Zr^2.5+^(4d^1.5^) and Os^2.6+^(5d^5.4^). In the case of Pb_2_HfOsO_6_, we assume the electron configurations are Hf^4+^(5d^0^:t^0^_2g_e^0^_g_) at S = 0 and Os^4+^(4f^14^5d^4^5s^0^:t^4^_2g_e^0^_g_) at S = 1. Then, calculation results provide that the valance states are Hf^2.4+^(4d^1.6^) and Os^2.6+^(5d^5.4^). With the GGA+U scheme, the valance states are also Hf^2.4+^(4d^1.6^) and Os^2.6+^(5d^5.4^), which are similar to the case under the GGA scheme. The valance states of Pb_2_VOsO_6_ could be represented by V^5+^ (3d^0^:t^0^_2g_e^0^_g_), S = 0 and Os^3+^(4f^14^5d^5^6s^0^:t^5^_g_e^0^_2g_) at S = 1/2. According to our calculation results, they imply the true valance states are V^1.5+^(3d^3.5^) and Os^2.6+^(5d^5.4^) based on the GGA method. With the GGA+U scheme, electrons of d orbital are 3.5 and 5.4 for V^1.5+^(3d^3.5^) and Os^2.6+^(5d^5.4^), suggesting that the distribution is identical to results with the GGA scheme.

In the cases of Pb_2_ZrOsO_6_, Pb_2_HfOsO_6_ and Pb_2_VOsO_6_, they not only have half-metallic property, but also boast narrower band gap when compared with other HMs in this paper. Under the GGA + U scheme, the gaps of compounds become wider; however, they are still remarkably smaller than those of others under the same scheme.

### 3.4. FiM-HM Compounds: Pb_2_XRhO_6_ (X = Zr and Hf)

In this group of compounds, there are two HM candidates, namely, Pb_2_ZrRhO_6_ and Pb_2_HfRhO_6_. With the GGA and GGA + U scheme, their final stable states belong to FiM rather than AF, as shown by the ΔE listed in Table 1. However, ΔE values of them are very small, at −8 and −10 meV/f.u. under the GGA scheme. The values of ΔE are too small to distinguish the preferred state of Pb_2_ZrRhO_6_ and Pb_2_HfRhO_6_, although they are negative. Nevertheless, the situation disappears while the calculation is in consideration of the GGA + U scheme. The results mean Pb_2_ZrRhO_6_ and Pb_2_HfRhO_6_ are considered as possible FiM-HM candidates.

Last, for Pb_2_ZrRhO_6_, the electron configurations are Zr^4+^(4d^0^:t^6^_2g_e^4^_g_) at S = 0 and Rh^4+^(4d^5^5s^0^: t^5^_2g_e^0^_g_) at S = 1/2. After calculation, the results showing the real valance states are Zr^2.5+^(4d^1.5^) and Rh^2.2+^(4d^6.8^) with the GGA scheme. With the GGA + U scheme, the distributions of electron number are Zr^2.5+^(4d^1.5^) and Rh^1.9+^(4d^7.1^). In the case of Pb_2_HfRhO_6_, the electron configurations we expected are Hf^4+^(5d^0^:t^0^_2g_e^0^_g_) at S = 0 and Rh^4+^(4d^5^5s^0^: t^5^_2g_e^0^_g_) at S = 1/2. Calculation results point out that the valance states of Pb_2_HfRhO_6_ are Hf^2.4+^(5d^1.6^) and Rh^2.3+^(4d^6.7^), while with the GGA + U scheme, the valance states are Hf^2.6+^(5d^1.4^) and Rh^2.1+^(4d^6.9^).

In the last group, the properties of Pb_2_ZrRhO_6_ and Pb_2_HfRhO_6_ studied here imply that they are the possible HM candidates. When the magnetic state is discussed, ΔE of Pb_2_ZrRhO_6_ and Pb_2_HfRhO_6_ are so small that we cannot determine whether they are in the FiM state or not under the GGA scheme. In consideration of the scheme with GGA + U, the ΔE decreases to −36 and −34 meV/f.u., and therefore we still categorize this group of compounds into FiM materials.

## 4. Conclusions

By the calculations with the GGA and GGA + U schemes, our work provides 13 possible FiM-HM candidates, namely Pb_2_NbTcO_6_, Pb_2_TaTcO_6_,Pb_2_TiRuO_6_, Pb_2_ZrRuO_6_, Pb_2_HfRuO_6_, Pb_2_VRuO_6_, Pb_2_NbRuO_6_, Pb_2_TaRuO_6_, Pb_2_ZrOsO_6_, Pb_2_HfOsO_6_, Pb_2_VOsO_6_, Pb_2_ZrRhO_6_ and Pb_2_HfRhO_6_. Then, we categorize these compounds in four groups according to X′ site element. We first analyze the case of the group of Pb_2_XTcO_6_. Omitting the small fluctuation of magnetic moment, Pb_2_TaTcO_6_ still remains with a possibility of being HM material when the strong correlation effect (GGA + U) is considered in the calculation, so it is still categorized in HM families. Fortunately, Pb_2_NbTcO_6_ retains its half-metallic property under the GGA + U scheme. Then, we also discuss the significant features for other groups. In the case of Pb_2_XRuO_6_, this group contains an abundance of HM candidates, which account for half of HM materials. Groups Pb_2_XOsO_6_, Pb_2_ZrOsO_6_, Pb_2_HfOsO_6_ and Pb_2_VOsO_6_ have a narrower band gap in comparison with other compounds. Compounds in the category of Pb_2_XRhO_6_ converge to an uncertain magnetic state (AF or FiM) and the ΔE decreases to −36 and −34 meV/f.u. under the scheme with GGA + U. Therefore, we still categorize this group of compounds into FiM materials. In short, the calculation within DFT provides 13 possible FiM-HM candidates, and we hope this result for the prediction of Pb-based double perovskites could provide systematic guidelines for future research on high-potential spintronics devices.

## Figures and Tables

**Table 1:** Physical properties of the selected FiM-HM family of Pb_2_XX′O_6_ (X = IVB/VB X′ = Rh, Hf, Os and Tc) in double perovskite structure by GGA and GGA + U calculations. The parenthesis behind U presents the on-site coulomb parameters: 3 eV for U_V_, and 2 eV for U_IVB_, U_Nb_, U_Ta_, U_Rh_, U_Hf_, U_Os_ and U_Tc_. (0,0) denotes the absence of GGA + U calculations. ΔE refers to the energy difference between FiM and AF states. The spin magnetic moments for X, X′ and the total moment are listed in the table as m_X_, m_X′_ and m_tot_, respectively. The number of electrons in the spin-up and spin-down orbitals is recorded for X(X′) element.

Materials Pb_2_XX′O_6_	$\left(U_{X},U_{X^{\prime }}\right)$	Spin Magnetic Moment (μ_B_/f.u.)			*d* Orbital Electrons ↑/↓		Band Gap (eV)	ΔE (meV/f.u.) FiM-AF
		m_X_	m_X′_	m_tot_	X	X′		
**NbTc**	(0,0)	−1.527	1.846	2.000	1.016/1.112	3.200/1.375	0.47/0.00	−21
	(2,2)	−1.886	1.980	2.000	0.913/1.034	3.251/1.292	0.95/0.00	−26
**TaTc**	(0,0)	−0.072	1.588	2.000	1.054/1.119	3.099/1.527	0.30/0.00	−96
	(2,2)	−0.116	1.691	2.011	1.004/1.115	3.141/1.467	0.80/0.00	−178
**TiRu**	(0,0)	−0.072	1.348	2.000	0.955/1.019	3.675/2.341	1.35/0.00	−43
	(2,2)	−0.098	1.378	2.000	0.916/1.008	3.691/2.329	1.80/0.00	−24
**ZrRu**	(0,0)	−0.031	1.386	2.000	0.747/0.771	3.696/2.325	1.18/0.00	−55
	(2,2)	−0.036	1.406	2.000	0.720/0.750	3.708/2.317	1.52/0.00	−106
**HfRu**	(0,0)	−0.028	1.379	2.000	0.784/0.802	3.690/2.326	1.25/0.00	−50
	(2,2)	−0.029	1.394	2.000	0.758/0.780	3.699/2.321	1.60/0.00	−92
**VRu**	(0,0)	−0.364	0.906	1.000	1.614/1.964	3.492/2.598	0.62/0.00	−18
	(3,2)	−0.875	1.174	1.000	1.341/2.193	3.613/2.452	1.23/0.00	−139
**NbRu**	(0,0)	−1.283	0.999	1.000	1.046/1.150	3.549/2.562	1.00/0.00	−49
	(2,2)	−1.531	1.052	1.000	0.960/1.081	3.578/2.538	1.30/0.00	−115
**TaRu**	(0,0)	−0.041	0.761	1.000	1.062/1.099	3.453/2.700	0.85/0.00	−22
	(2,2)	−0.051	0.767	1.000	1.029/1.077	3.466/2.706	1.12/0.00	−60
**ZrOs**	(0,0)	−0.030	1.372	2.000	0.746/0.772	3.386/2.032	0.38/0.00	−27
	(2,2)	−0.044	1.462	2.000	0.714/0.756	3.442/1.998	0.75/0.00	−112
**HfOs**	(0,0)	−0.025	1.372	2.000	0.782/0.800	3.386/2.032	0.47/0.00	−28
	(2,2)	−0.032	1.452	2.000	0.752/0.779	3.436/2.002	0.82/0.00	−97
**VOs**	(0,0)	−0.837	1.218	1.000	1.366/2.182	3.310/2.109	0.65/0.00	−30
	(3,2)	−1.457	1.629	1.000	1.050/2.475	3.492/1.884	1.65/0.00	−309
**ZrRh**	(0,0)	−0.021	0.557	1.000	0.752/0.769	3.700/3.147	1.60/0.00	−10
	(2,2)	−0.021	0.529	1.000	0.728/0.746	3.968/3.172	1.82/0.00	−36
**HfRh**	(0,0)	−0.020	0.548	1.000	0.788/0.802	3.695/3.150	1.57/0.00	−8
	(2,2)	−0.019	0.517	1.000	0.764/0.779	3.690/3.175	1.82/0.00	−34

**Table 2:** In the table below, total energy of possible HM candidates with each magnetic state is presented. U_X(X′)_ are the effective parameters used in GGA + U calculations for X(X′).

Materials Pb_2_XX′O_6_	$\left(U_{X},U_{X^{\prime }}\right)$	Final States	E (eV/f.u.)	Materials Pb_2_XX′O_6_	$\left(U_{X},U_{X\prime }\right)$	Final States	E (eV/f.u.)
**NbTc**	(0,0)	AF	−74.269	**TaRu**	(0,0)	AF	−74.610
	(2,2)	AF	−72.512		(2,2)	AF	−72.606
	(0,0)	FiM	−74.290		(0,0)	FiM	−74.632
	(2,2)	FiM	−72.538		(2,2)	FiM	−72.666
**TaTc**	(0,0)	AF	−76.443	**ZrOs**	(0,0)	AF	−73.595
	(2,2)	AF	−74.486		(2,2)	AF	−71.755
	(0,0)	FiM	−76.539		(0,0)	FiM	−73.622
	(2,2)	FiM	−74.664		(2,2)	FiM	−71.867
**TiRu**	(0,0)	AF	−70.600	**HfOs**	(0,0)	AF	−75.677
	(2,2)	AF	−68.628		(2,2)	AF	−73.851
	(0,0)	FiM	−70.643		(0,0)	FiM	−75.705
	(2,2)	FiM	−68.752		(2,2)	FiM	−73.948
**ZrRu**	(0,0)	AF	−71.763	**VOs**	(0,0)	AF	−71.377
	(2,2)	AF	−69.993		(3,2)	AF	−67.913
	(0,0)	FiM	−71.818		(0,0)	FiM	−71.407
	(2,2)	FiM	−70.099		(3,2)	FiM	−68.222
**HfRu**	(0,0)	AF	−73.897	**ZrRh**	(0,0)	AF	−69.142
	(2,2)	AF	−72.119		(2,2)	AF	−67.422
	(0,0)	FiM	−73.947		(0,0)	FiM	−69.152
	(2,2)	FiM	−72.211		(2,2)	FiM	−67.458
**VRu**	(0,0)	AF	−69.729	**HfRh**	(0,0)	AF	−71.277
	(3,2)	AF	−66.201		(2,2)	AF	−69.542
	(0,0)	FiM	−69.747		(0,0)	FiM	−71.285
	(3,2)	FiM	−66.340		(2,2)	FiM	−69.576
**NbRu**	(0,0)	AF	−72.123				
	(2,2)	AF	−70.084				
	(0,0)	FiM	−72.172				
	(2,2)	FiM	−70.199				

## Data Availability

The data supporting this study’s findings are available from the corresponding author upon reasonable request.
